# Combined effects of hyperthermia and chemotherapy on the regulate autophagy of oral squamous cell carcinoma cells under a hypoxic microenvironment

**DOI:** 10.1038/s41420-021-00538-5

**Published:** 2021-08-31

**Authors:** Fan Shi, Dan Luo, Xuexiao Zhou, Qiaozhen Sun, Pei Shen, Shengzhi Wang

**Affiliations:** 1 School of Stomatology of Qingdao University, Qingdao, China; 2 Department of Oral and Maxillofacial Surgery, The Affiliated Yantai Yuhuangding Hospital of Qingdao University, Yantai, China; 3 Institute for Translational Medicine, Department of Stomatology, The Affiliated Hospital of Qingdao University, Qingdao, China

**Keywords:** Oral cancer, Preclinical research

## Abstract

Autophagy has a complex dual role in tumor survival or cell death owning to that is an evolutionarily conserved catabolic mechanism and provides the cells with a sustainable source of biomolecules and energy for the maintenance of homeostasis under stressful conditions such as tumor microenvironment. Hyperthermia is a rapidly growing field in cancer therapy and many advances have been made in understanding and applying the mechanisms of hyperthermia. The shallow oral and maxillofacial position and its abundant blood supply are favorable for the use of hyperthermia. However, the relationship between hyperthermia and autophagy has not been examined of oral squamous cell carcinoma (OSCC) in the tumor hypoxia microenvironment. Here, the expression level of autophagy relative genes is examined to explore autophagy effect on the responses of hyperthermia, hypoxia, and innutrition tumor microenvironment. It is founded that hyperthermia and hypoxia cause autophagy in starvation conditions; further, in hypoxia and innutrition tumor microenvironment, hyperthermia combines YC-1 and 3-MA could inhibit HIF-1α/BNIP3/Beclin1 signal pathway and decrease the secretion of HMGB1; moreover, the cell apoptosis rate increases with an inhibited of cell migration capacity. Thus, the present study demonstrated that combined use of YC-1 and 3-MA might increase the death of tumor cells in physiological and hyperthermic conditions, which could be relevant with the inhibition of autophagy in OSCC tumor cells under hypoxia microenvironment in vitro, which offers new insight into the therapy of OSCC and its application in treating others study carcinomas.

## Introduction

Oral squamous cell carcinoma (OSCC) accounts for more than 95% of all head and neck cancer and is an aggressive group of tumors characterized by high rates of regional lymph node metastasis and local recurrence, less than 50% of patients will survive for 5 years [1]. OSCC often leads to patients with maxillofacial deformities and affects patients’ esthetics and quality of life. The mainstays of therapeutic schedules for OSCC comprises surgery, radiotherapy, chemotherapy, or a combination of these modalities depending on the extent of the disease, which is associated with a substantial morbidity and toxicity rate [2, 3]. However, the strategies above have some defects, conventional surgical affects maxillofacial morphology and esthetic, chemotherapy exhibits many side-effects leading to an increase in the toxicity profile among OSCC patients. OSCC as a solid tumor, cancer cells proliferate rapidly and form large solid tumor masses, leading to obstruction and compression of the blood vessels surrounding these masses these abnormal blood vessels do not function sufficiently and generate poor O_2_ supply to the central tumor regions, and the levels of oxygenation within the same tumor are highly variable from one area to another and can change over time [4, 5], therefore, hypoxia (Hy) is a crucial microenvironment condition for tumor pathophysiology and tumor metastasis. As an adaptive response to hypoxic stress, hypoxic tumor cells activate several survival pathways to carry out their essential biological processes compared with normal cells. The hypoxia-inducible factor-1α (HIF-1α) is one crucial facilitator for energy adaption and oxygen metabolic stress in hypoxia and nutrition deficiency tumor environment, and functions as a general regulator of tumor aggressiveness and metastasis as well [4, 6, 7].

Hyperthermia (HT) is a potent radiosensitizer, which sensitizes tumor cells to radiation by inhibiting DNA repair and increasing the aggregation of damaged nuclear proteins [8, 9]. Furthermore, hyperthermia-related elevated blood flow and vascular permeability in the heated tumor region also promote higher intratumor and peritumor drug concentrations to improve the efficacy of chemotherapy [9–11]. And which in combination with radiotherapy, enforces immunomodulation akin to “in situ tumor vaccination” [12]. Therefore, hyperthermia acts as a complement of radiotherapy, chemotherapy, and molecular targeted monotherapy.

Autophagy is a cellular pathway that presents only in eukaryotic cells to degrade the aged and damaged organelles as well as misfolded proteins. It functions as a recycling program to provide biofuel to cells from degraded macromolecules to maintain sufficient ATP production for survival and is a key component in maintaining homeostasis of the cellular environment [13]. Depending on the exact cell type and conditions, it either acts as a protagonist or an antagonist of apoptosis [14]. Autophagy also plays a crucial role in cancer pathophysiology. It is believed to prevent cancer development in normal tissue, but when in the severe tumor microenvironment of solid malignancies, as an adaptive response, which helps cells recycle metabolites and organelles in order to survive and protect cancer cells within an already established tumor from the shortage of nutrients and hypoxic conditions [14, 15]. Previous studies have shown that initiating triggers for death in heat-shocked cells include induction of physiological cascades; thermal protein unfolding and aggregation; necrosis that occurred at extremely elevated temperatures [16]. The ability of hyperthermia to augment radiation therapy and chemotherapy has been demonstrated in clinical oncology as evidenced in many phases II and III trials [17, 18]. However, tumor cells possess homeostatic responses to reduce heat-shock-induced cell death, which involves cell cycle arrest and transient induction of the transcription of genes encoding molecular chaperones and heat shock proteins (HSPs) [19]. In short, hyperthermia induces the expression of HSPs and inhibits DNA damaged to repair, whereas DNA damage, hyperthermia, and HPSs evoke autophagy, which was associated with facilitated cell survival and decreased programmed cell death [16, 19–21]. Furthermore, cellular damage caused by heating can be repaired and reversed by autophagy, resulting in incomplete cell necrosis [21, 22]. HMGB1 is a late inflammatory mediator associated with sepsis, malignancy, and immune disease [23], which is passively released by necrotic tissues or actively secreted by stressed cells [24]. Intracellularly, HMGB1 is involved in DNA repair, transcription, and recombination as well in the regulation of apoptosis/autophagy balance. Once secreted, it participates in a variety of processes such as inflammation, proliferation, differentiation, migration, invasion, and tissue regeneration [25]. Heat shock stress from hyperthermia can lead to cell necrosis, and literature reported necrosis-inducing anticancer drugs to enhance high mobility group box-1 protein (HMGB1) released during cell necrosis [26], and HMGB1 regulates autophagy [23].

Therefore, HSPs and autophagy are two controllers of cellular proteostasis. Under stressful cellular conditions, such as hyperthermia, hypoxia, and energy deficiency, these two mechanisms are likely to complement each other [16]. However, it remains unknown whether hyperthermia-induced autophagy facilitates cell survival or accelerates cell death in hypoxia and nutrition-deficient tumor microenvironment of human OSCC. Hence, we aimed to investigate the relationship between hyperthermia and autophagy in hypoxia and nutrition-deficient tumor microenvironment of human OSCC. Meanwhile, the underlying mechanism was examined. This study might provide a novel promising therapeutic regiment for human OSCC.

## Results

### HIF-1α and Beclin1 expression levels increased in OSCC tissues

**Fig. 1:**
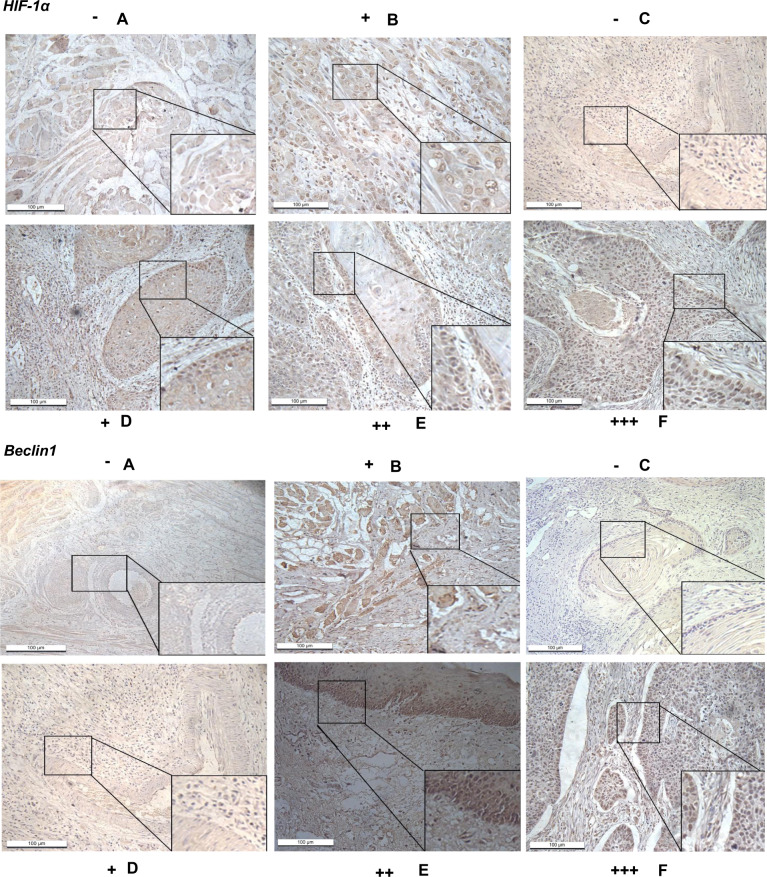
The expression level of HIF-1α and Beclin1 in OSCC tissues and para cancer tissues (×200) HIF-1α was mainly expressed in the cell nucleus while Beclin1 was mainly located in the cell membrane and cytoplasm with a small amount in the cell nucleus. The positive staining of HIF-1α and Beclin1 showed uniform light yellow, brownish-yellow, or tan granules. **A**, **B** Normal tissue. **C**–**F** OSCC tissues. HIF-1α hypoxia-inducible factor-1α, Beclin1, a coiled-coil Bcl-2-interacting protein homologous, OSCC oral squamous cell carcinoma.

**Fig. 2:**
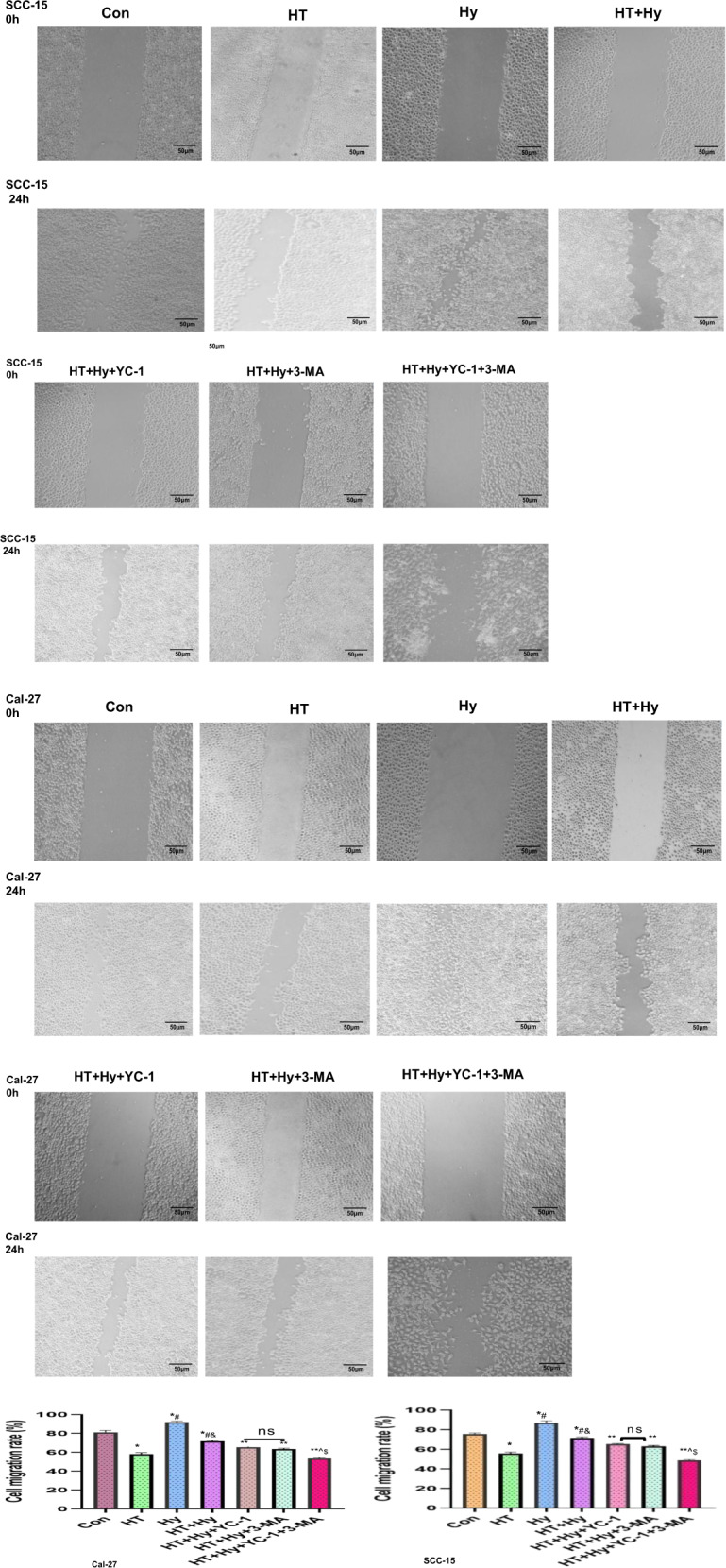
HT combined YC-1, 3-MA alone or both inhibited the migration of Cal-27 and SCC-15 cells in hypoxia and starvation microenvironment, as determined by wound healing assay. Magnification, ×400. Data were expressed as the mean ± SEM from three independent experiments. Con control, HT hyperthermia, Hy hypoxia; YC-1: 3-(5′-hydroxymethyl-2′-furyl)−1-benzylindazole; 3-MA: 3-Methyladenine; the histogram of cell migration rate after treatments were analyzed. *p < 0.05 vs. Con group; #*p* < 0.05 vs. HT group; &*p* < 0.05 vs. Hy group; **p < 0.05 vs. HT + Hy group; ^*p* < 0.05 vs. HT + Hy+3-MA group; $*p* < 0.05 vs. HT + Hy + YC-1 group; ns no significant difference (*p* > 0.05).

The clinical significance of HIF-1α and Beclin1 in OSCC patients was evaluated by analyzing the expression level of HIF-1α and Beclin1 in 80 OSCC tissues and pare-cancer tissue (tumor margin adjacent normal tissues) by the scoring of IHC (Fig. 1). HIF-1α is mainly located in the cell nucleus while Beclin1 is mainly located in the cell membrane and cytoplasm with a small amount in the cell nucleus. In this study, we observed that HIF-1α and Beclin1 were expressed both in cancer tissue and adjacent normal tissues. By multiplying the score of staining intensity and percentage of positive cells of each tissue section, we found that HIF-1α and Beclin1 were both highly expressed in OSCC tissues compared with pare-cancer tissue (tumor margin adjacent normal tissues) (*p* < 0.05, Supplementary Table 1). Besides, the high expression level of HIF-1α and Beclin1 was associated with poor cell differentiation, lymph node metastasis, advanced pathological TNM stage, and large tumor size (*p* < 0.05, Table 1), but was not correlated with gender or age (Fig. 2).

**Table 1:** Association between HIF-1α, Beclin1 expression, and the clinical as well histopathological features of patients with oral squamous cell carcinoma (*n* = 80).

Variables	Number of patients (*n*)	HIF-1a positive (*n*)	HIF-1a positive rate (%)	χ^2^	*p*	Beclin1 positive (*n*)	Beclin1 positive rate (%)	χ^2^	*p*
Sex									
Male	56	39	69.6%	0.069	0.966	39	69.64%	1.372	0.504
Female	24	16	66.67%			19	79.17%		
Age, years									
≥60	55	47	85.45%	1.064	0.587	43	78.18%	0.9503	0.622
<60	25	19	76%			17	68%		
Tumor size, cm									
≥1.5	53	43	81.13%	6.661	0.037	42	79.25%	10.87	0.004
<1.5	27	14	51.85%			11	40.74%		
TNM stage									
I or II	24	14	58.3%	8.54	0.015	11	45.83%	8.412	0.015
III or IV	56	49	87.5%			44	78.57%		
Differentiation									
Well	29	13	44.83%	8.137	0.017	12	41.38%	6.572	0.037
Moderate/poor	51	39	76.47%			36	70.59%		
Lymph node metastasis									
Positive	47	39	82.98%	33.71	0.001	33	70.21%	6.729	0.035
Negative	33	7	21.21%			14	42.42%		

### Establishment of hypoxic microenvironment

**Fig. 3:**
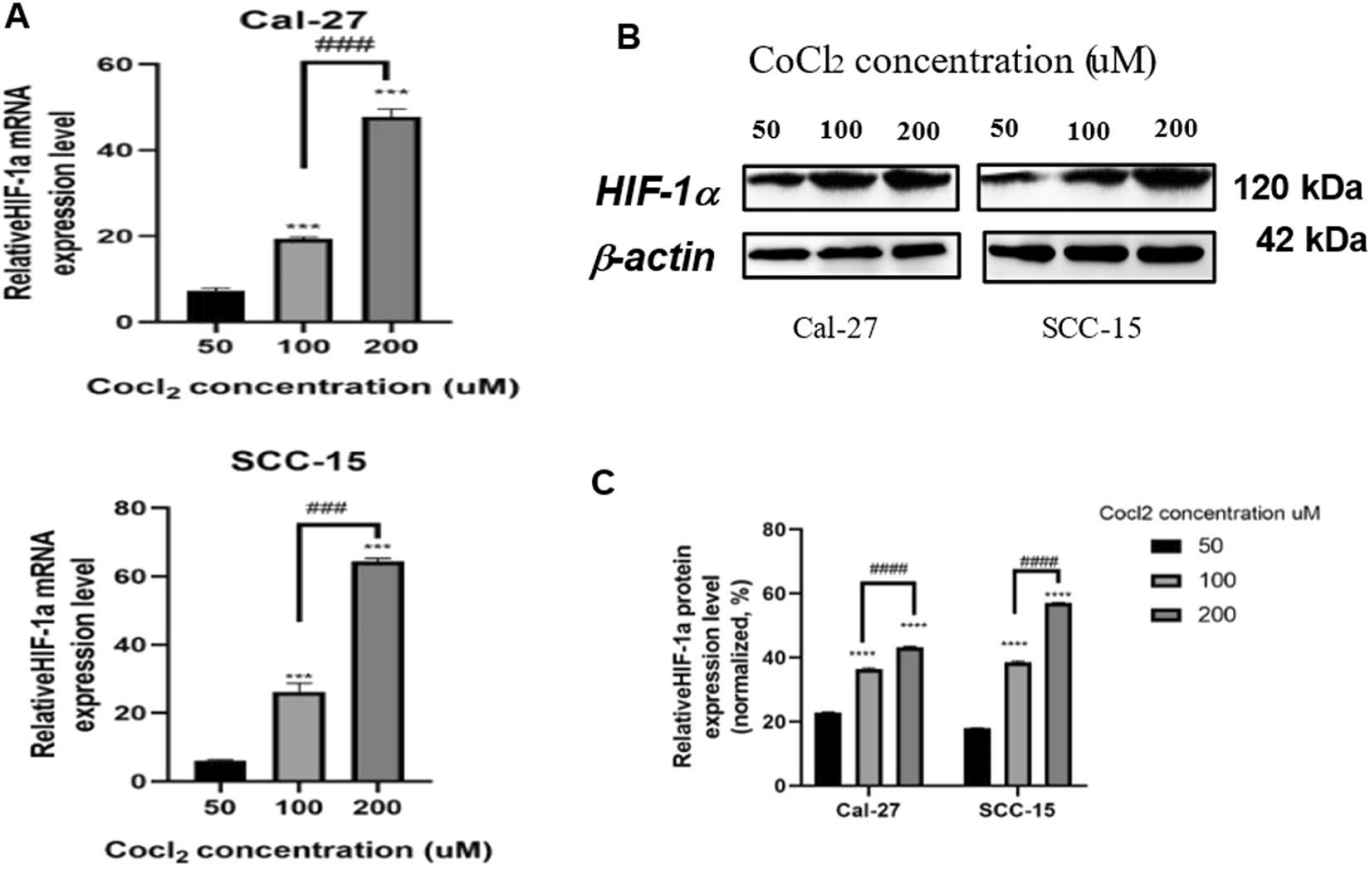
CoCl_2_ induced the mRNA and protein expression of HIF-1*α* to construct a hypoxic microenvironment. CoCl_2_ significantly induced the mRNA (**A**) and protein (**B**) and (**C**) expression of HIF-1α in a dose-dependent manner. Cal-27 and SCC-15 cells were co-cultured with different concentrations of CoCl2 for 24 h, then the mRNA and protein expression levels of HIF-1α were analyzed by qRT-PCR and Western blot, which were normalized to those of β-actin. Data were expressed as the mean ± SEM from three independent experiments. *p < 0.05, **p < 0.01, ***p < 0.001 vs 50 μM CoCl_2_; #*p* < 0.05, ##*p* < 0.01, ###*p* < 0.001 vs. 50 μM CoCl_2_.

Low oxygen-induced hypoxia is the optimal hypoxia model. However, induction of chemical hypoxic conditions using CoCl_2_ allows the researcher to open the culture container many times while maintaining a stable level of HIF-1α. The IC50 values for CoCl_2_ in Cal-27 and SCC-15 cells were 108.3 μM and 99.68 μM, respectively, as determined by the CCK-8 cell viability assay. Therefore, we used 100 μM CoCl_2_ in free-serum RPIM 1640 to achieve the hypoxia and starvation microenvironment for the subsequent experiments (Supplementary Table 3 and Fig. S1). Our results showed that compared with the untreated control group, stable mRNA and protein expression of HIF-1α could be induced in Cal-27 and SCC-15 cells in a dose-dependent manner by CoCl_2_ under normoxia, as measured by qRT-PCR and Western blot respectively, which demonstrated the successful establishment of the hypoxic tumor microenvironment (Fig. 3A–C). Compared with the control group, Cal-27 and SCC-15 cells both presented a significant augment in cell migration under the hypoxia microenvironment (*p* < 0.05, Fig. 2). This result suggested that the hypoxia microenvironment facilitated HIF-1α expression and promoted cell migration. In general, the results from the two cell lines (Cal-27 and SCC-15) showed a similar tendency.

### Hyperthermia induced autophagy of OSCC cells in the hypoxic microenvironment

**Fig. 4:**
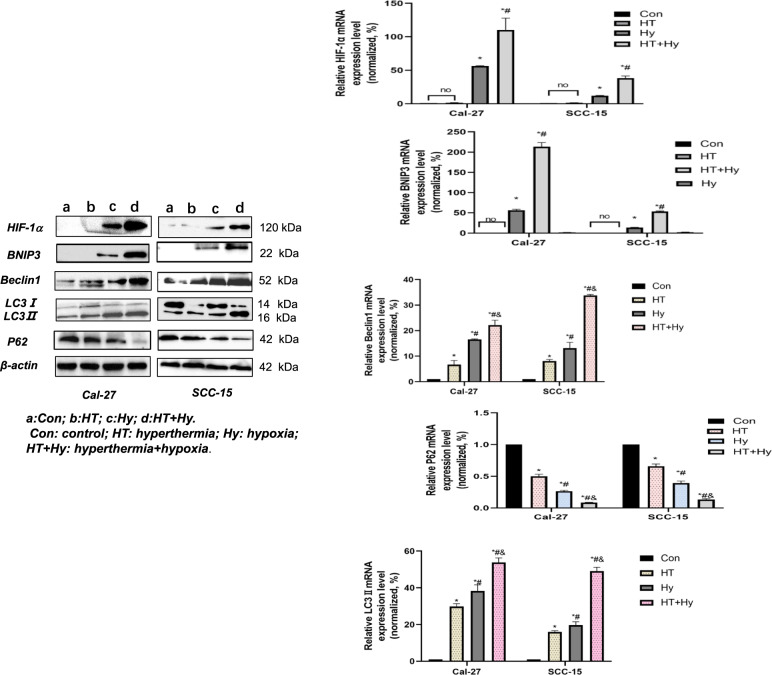
Hyperthermia and hypoxia-induced the expression of autophagy hallmark proteins in OSCC cells. Cal-27 and SCC-15 cells were cultured in hypoxia conditions and were exposed to 42 °C for 1 h, followed by recovery at 37 °C for 24 h. The protein and mRNA levels of HIF-1α, BNIP3, Beclin1, LC3-II, and p62 were analyzed by qRT-PCR and western blot and normalized to those of β-actin. Data were expressed as the mean ± SEM from three independent experiments. a: Con; b: HT; c: Hy; d: HT + Hy. Con control, HT hyperthermia, Hy hypoxia, HT + Hy hyperthermia+hypoxia. *p < 0.05 vs. Con group; #*p* < 0.05 vs. HT group; &*p* < 0.05 vs. Hy group; no: no significant difference (*p* > 0.05).

To clarify the relationship between hypoxia, hyperthermia, and autophagy, we tested proteins related to autophagy signaling pathway by Western blot and qRT-PCR in Cal-27 and SCC-15 cells, and found that HIF-1α and BNIP3 were hardly expressed in the untreated control group and “HT” group (hyperthermia treatment alone), and there was no statistical difference in the expression of HIF-1α and BNIP3 in HT group compared the control group (*p* > 0.05, Fig. 4, Supplementary Fig. S2). This indicated that HIF-1α and BNIP3 were not expressed in abundance under normal oxygen conditions, and hyperthermia could not induce the expression of HIF-1α and BNIP3 in normoxia conditions. The expression of autophagy-related genes such as Beclin1 and LC3II were higher in the HT group compared to the control group (*p* < 0.05, Fig. 4, Supplementary Fig. S2), showing that Beclin1 and LC3II were expressed under constant oxygen condition and hyperthermia promoted Beclin1 and LC3II expression. Besides, the expression of P62 protein sharply decreased in the HT group compared to the control group (*p* < 0.05, Fig. 4, Supplementary Fig. S2). Taken together, the results showed that hyperthermia could induce autophagy under normal oxygen conditions. However, the expression level of HIF-1α, BNIP3, Beclin1, LC3II significantly increased in the Hy group (hypoxia treatment alone) and the “HT + Hy” group (combined hypoxia and hyperthermia treatment) compared to the control group and HT group (*p* < 0.05). On contrary, the expression of P62 sharply decreased in the Hy group and “HT + Hy” group compared to the control group and HT group (*p* < 0.05), what is more, we found that compared with the Hy group, the expression of HIF-1α, BNIP3, Beclin1, LC3II were significantly higher, whereas that of p62 proteins were remarkably lower in the “HT + Hy” group (*p* < 0.05, Fig. 4, Supplementary Fig. S2). In general, the results from the two cell lines (Cal-27 and SCC-15) showed a similar tendency.

### Hyperthermia combined chemotherapy inhibited autophagy

**Fig. 5:**
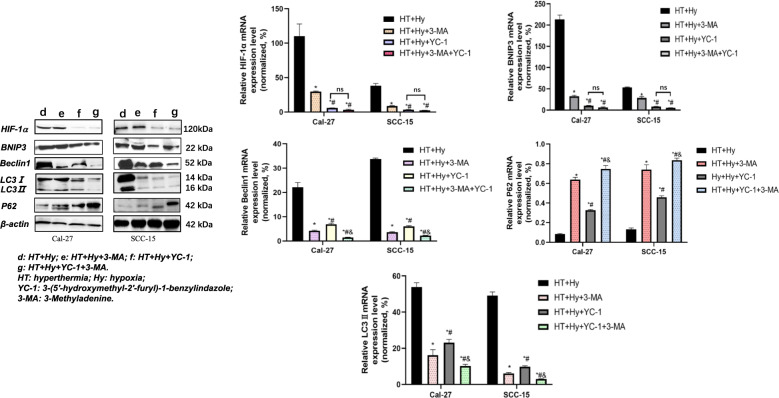
The addition of YC-1 and 3-MA inhibited HT-induced autophagy in hypoxia and starvation tumor microenvironment, both of which involved downregulation of the HIF-1α/BNIP3 /Beclin1 signaling pathway. The mRNA and protein expression of HIF-1α, BNIP3, Beclin1, LC3-II, and p62 in OSCC cells were measured by Western blot and qRT-PCR, which were normalized to β-actin. The results were presented as the mean ± standard deviation of three independent experiments. d: HT + Hy; e: HT + Hy+3-MA; f: HT + Hy+YC-1; g: HT + Hy+YC-1 + 3-MA. HT: hyperthermia; Hy: hypoxia; YC-1: 3-(5′-hydroxymethyl-2′-furyl)−1-benzylindazole; 3-MA: 3-Methyladenine. *p < 0.05 vs. HT + Hy group; #*p* < 0.05 vs. HT + Hy+3-MA group; &*p* < 0.05 vs. HT + Hy+YC-1 group; ns no significant difference (*p* > 0.05).

To further investigate the correlation between hyperthermia-induced the HIF-1α/BNIP3/Beclin1 autophagy signaling pathway in hypoxia and starvation tumor microenvironment, YC-1 was used to suppress the HIF-1α-mediated autophagy pathway, and 3-MA was used for direct inhibition of autophagy. First, the IC50 of YC-1 and 3-MA was assessed by CCK-8. The IC50 values for YC-1 in Cal-27 and SCC-15 cells were 24.37 μM and 34.72 μM, respectively (Supplementary Fig. S1, Table 4). We used 20 μM for Cal-27 and 30 μM for SCC-15 cells to conduct the following experiments. The IC50 values for 3-MA in Cal-27 and SCC-15 cells were 35.77 μM and 30.89 μM, respectively (Supplementary Fig. S1, Table 5), therefore we used 35 μM (Cal-27 cell) and 30 μM (SCC-15 cell) in the subsequent experiments. Through PCR and Western blot analysis we found that the expression levels of HIF-1α and BNIP3 were not statistically different in the “HT + Hy” group vs. the “HT + Hy+3-MA” group and “HT + Hy+YC-1” group vs. “HT + Hy+YC-1 + 3-MA” group (*p* < 0.05, Fig. 5, Supplementary Fig. S3). Both HIF-1α and BNIP3 expressions were lower in the “HT + Hy+YC-1” group than in the “HT + Hy” group and “HT + Hy+3-MA” group and were even lower in the “HT + Hy+YC-1 + 3-MA” group. Reduced Beclin1 and LC3II expression levels were observed in the following groups: HT + Hy+YC-1 + 3-MA < HT + Hy+3-MA < HT + Hy+YC-1 < HT + Hy. Conversely, increased expression levels of P62 were observed in the following groups: HT + Hy+YC-1 + 3-MA > HT + Hy+3-MA > HT + Hy+YC-1 > HT + Hy (*p* < 0.05, Fig. 5, Supplementary Fig. S3). These data indicated hyperthermia-induced autophagy through activating the HIF-1α/BNIP3/Beclin1 signaling pathway. 3-MA had a stronger effect in inhibiting autophagy than YC-1, and combined use of 3-MA and YC-1 provided further inhibition of autophagy in OSCC cells. Therefore, we concluded that hyperthermia might not only induce autophagy through activating the HIF-1α/BNIP3/Beclin1 signaling pathway but also involve other pathways in the hypoxia and starvation microenvironment. Moreover, hyperthermia combined with chemotherapy inhibits autophagy. In general, the results from the two cell lines (Cal-27 and SCC-15) showed a similar tendency.

### Hyperthermia combined chemotherapy inhibited the secretion of HMGB1

**Fig. 6:**
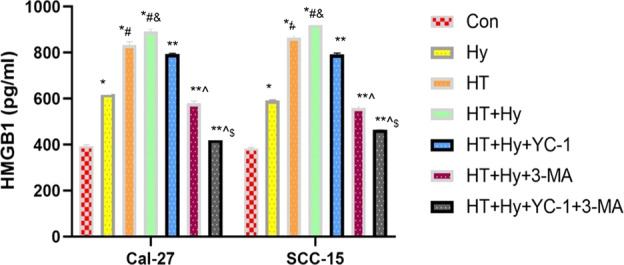
HT combined YC-1, 3-MA alone or both inhibited the secretion of HMGB1 in Cal-27 and SCC-15 cells under hypoxia and starvation microenvironment as measured by ELISA. Data were presented as mean ± SEM from three independent experiments. Con control, HT hyperthermia, Hy hypoxia, YC-1: 3-(5′-hydroxymethyl-2′-furyl)-1-benzylindazole; 3-MA:3-methyladenine; *p < 0.05 vs. Con group; #*p* < 0.05 vs. HT group; &*p* < 0.05 vs. Hy group; **p < 0.05 vs. HT + Hy group; ^*p* < 0.05 vs. HT + Hy + 3-MA group; $*p* < 0.05 vs. HT + Hy + YC-1 group; ns no significant difference (*p* > 0.05).

HMGB1 was translocated from the nucleus to the cytoplasm and secreted or passively released through the permeabilized plasma membrane of succumbing/dead cells. We measured the extracellular HMGB1 protein by ELISA and found that compared with the control group, the secretion of HMGB1 significantly increased with hypoxia or hyperthermia treatment alone and combined in Cal-27 and SCC-15 cells, with the protein level in the “HT + Hy” group > HT group > Hy group > control group (*p* < 0.05, Fig. 6), suggesting that hyperthermia and hypoxia promoted HMGB1 secretion under normal oxygen conditions and that hyperthermia was stronger than hypoxia in promoting HMGB1 secretion. On the contrary, the addition of 3-MA and YC-1 significantly reduced the secretion of HMGB1, with protein level in the “HT + Hy+YC-1 + 3-MA” group < “HT + Hy+3-MA” group < “HT + Hy+YC-1” group < “HT + Hy” group (*p* < 0.05, Fig. 6). Based on the above experimental data, we concluded that hyperthermia and hypoxia might facilitate HMGB1 secretion in the starvation tumor microenvironment, and the use of chemotherapy drugs could also inhibit the secretion of HMGB1 in addition to autophagy inhibition. Therefore, we speculated that there is also a connection between HMGB1 secretion and autophagy. In general, the results from the two cell lines (Cal-27 and SCC-15) showed a similar tendency.

### Inhibition of autophagy reduced tumor cell migration in hypoxia and starvation microenvironment

To determine the effect of autophagy and hypoxia on Cal-27 and SCC-15 cell migration, we performed a wound-healing assay in Cal-27 and SCC-15 cells and the results showed compared with untreated control cells, cell mobility increased in the Hy group and decreased in the HT group, which demonstrated hypoxia might promote cell migration while hyperthermia inhibits cell migration (*p* < 0.05, Fig. 2). In addition, the “HT + Hy” group had worse migratory ability than the “Hy” group, which demonstrated that hyperthermia could inhibit cell migration whether in normoxia or hypoxia conditions. Compared with the “HT + Hy” group, the cell migration of the “HT + Hy+3-MA” group, “HT + Hy+YC-1” group, and “HT + Hy+YC-1 + 3-MA” group were all significantly reduced (*p* < 0.05, Fig. 2), and the “HT + Hy+YC-1 + 3-MA” group had the strongest inhibition of cell migration, which indicated that inhibiting autophagy might reduce tumor cell migration in hypoxia microenvironment. However, there was no significant difference in migration between the “HT + Hy + 3-MA” group and the “HT + Hy+YC-1” group (*p* *>* 0.05, Fig. 2). In general, the results from the two cell lines (Cal-27 and SCC-15) showed a similar tendency.

### Inhibition of autophagy enhanced hyperthermia-induced apoptosis in OSCC cells

**Fig. 7:**
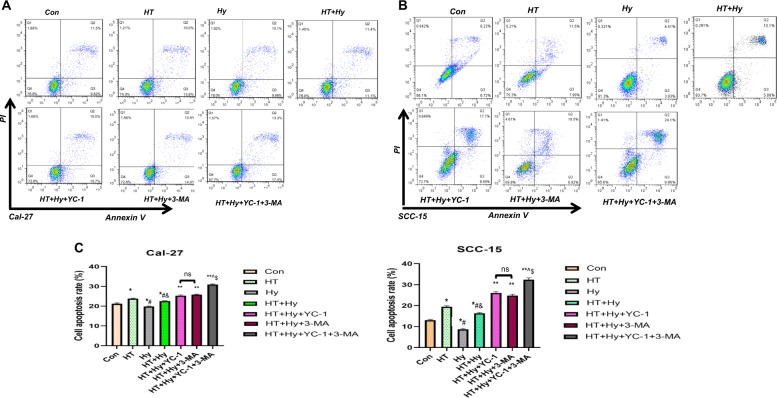
HT combined YC-1, 3-MA alone or both facilitated the apoptosis of Cal-27 and SCC-15 cells in hypoxia and starvation microenvironment, as measured by flow cytometry. Data were presented as the mean ± SEM from three independent experiments. Con control, HT hyperthermia, Hy hypoxia, YC-1: 3-(5′-hydroxymethyl-2′-furyl)−1-benzylindazole; 3-MA: 3-Methyladenine. The histogram of cell apoptosis rate after treatments were analyzed. *p < 0.05 vs. Con group; #*p* < 0.05 vs. HT group; &*p* < 0.05 vs. Hy group; **p < 0.05 vs. HT + Hy group; ^*p* < 0.05 vs. HT + Hy + 3-MA group; $*p* < 0.05 vs. HT + Hy + YC-1 group; ns no significant difference (*p* > 0.05).

Apoptosis was investigated using flow cytometry analysis. We found that compared with the control group, the cell apoptosis rate increased in the Hy group and decreased in the HT group. Meanwhile, the cell apoptosis rate of the “HT + Hy” group was higher than the Hy group and control group (*p* < 0.05, Fig. 7), which supported the conclusion that hyperthermia significantly promoted tumor cell apoptosis at normal oxygen concentration and hypoxia condition, while hypoxia inhibited cell apoptosis in nutrition deficiency microenvironment. Besides, YC-1 and 3-MA both facilitated cell apoptosis in approximately equivalent scale (*p* > 0.05) and combined use of both drugs had the strongest effect on cell apoptosis (*p* < 0.05, Fig. 7). However, in terms of the apoptotic sensitivity of Cal-27 and SCC-15 cell lines, compared with the SCC-15 cell line, the apoptotic sensitivity of the Cal-27 cell line was lower under various treatment conditions, which may result in the cell lines have a certain resistance to various treatments. In general, the results from the two cell lines (Cal-27 and SCC-15) showed a similar tendency, namely, HT combined with chemotherapy could significantly promote OSCC cell apoptosis by inhibiting autophagy under hypoxia and nutrition deficiency microenvironment in vitro.

## Discussion

OSCC is the most common solid tumor in the head and neck, and a growing body of evidence indicates that the microenvironment of a solid tumor with hypoxia, low PH, poor nutrition, poor perfusion, and abnormally high interstitial fluid pressure change the biological behavior of the tumor, which significantly reduces the effect of radiotherapy and chemotherapy and promotes tumor cell proliferation, invasion, and migration [27, 28]. The adaptation and survival of tumor cells in heterogeneous microenvironments requires the coordination of complex pathways and mechanisms, such as hypoxia induction factor 1 (HIF-1), unfolded protein reaction (UPR), rapamycin (mTOR), and autophagy [29]. Hypoxia is a crucial microenvironment condition for solid tumor pathophysiology, HIF-1α is a key molecule that is highly expressed under hypoxia. In general, under the condition of normoxia, the biosynthesis of mitochondrial respiration and anabolism is facilitated by oncoprotein *MYC*, allowing cancer cells to proliferate under conditions of adequate oxygen and nutrition [30, 31]. But under hypoxia, energy metabolism is regulated by HIF-1 rather than MYC, HIF-1 is a powerful mediator of carbohydrate reprogramming from oxidative phosphorylation to glycolytic metabolism in hypoxic reactions by regulating oxygen transport (angiogenesis) and oxygen consumption (glycolysis metabolism) [32]. Metabolic pathway changes of tumor cells during hypoxia or malnutrition are considered to be a feature of cancer cells, namely metabolic reprogramming [33]. Also, when tumor cells are exposed to a stressful microenvironment, especially low PH, low oxygen, and nutrient deficiency, autophagy is activated to cycle cellular metabolic substrate to meet their high metabolism and energy demand, as well as to suppress the body’s inflammatory response induced by the tumor to prevent the cytotoxicity accumulation and to promote tumor cell survival. Therefore, autophagy constitutes a way to prolong the survival of tumor cells [27, 28, 34, 35]. Therefore, HIF-1α is considered to be a potential prognostic marker of many cancers, including OSCC [36]. The formation of HIF-1α is oxygen-dependent. At the tumor margin, blood vessels grow to provide sufficient nutrients and oxygen, allowing the synthesis and rapid ubiquitin-mediated degradation within 10 min of HIF-1α under normoxia. In contrast, HIF-1α protein activity is prolonged under hypoxia [30]. Therefore, one important consequence of hypoxia is the induction of HIF-1α, which activates a series of downstream genes that facilitate tumor cell survival in the hypoxia microenvironment, such as autophagy-related genes Beclin1 [37]. In this study, we detected the expression level of HIF-1α and Beclin1 in 80 pairs of OSCC tissues and adjacent normal tissue, and our results suggested that HIF-1α and Beclin1 were both highly expressed in OSCC tissues compared with normal tissues and were significantly associated with large tumor size, advanced TNM grade, high pathological grade and lymphocytic infiltration. This finding is consistent with Ribeiro et al. research results [38].

Kinds of literature have reported that autophagy is a self-degrading process and plays an indispensable role in sustaining cellular homeostasis under stress [39, 40]. The role of autophagy in cancer is most dramatic and dynamic. In normal cells, autophagy inhibits tumor occurrence, however, in hypoxia and nutrient deficiency tumor microenvironment, autophagy promotes tumor cell survival as an alternative energy supply pathway besides the “Warburg effect”. Autophagy initiation occurs under stress conditions, such as nutrient and energy deficiency, hypoxia, reactive oxygen species (ROS), protein aggregation, and production of damaged organelles [41, 42]. As is well known, the conversion of cytosolic LC3-I to LC3-II, which binds autophagic vacuoles, is the primary hallmark of autophagy. The protein p62 (also known as SQSTM1) has been reported to interact with the autophagic effector protein LC3 and to be degraded through an autophagy-lysosome pathway [43]. Therefore, activation of autophagic flux leads to a decrease in p62 and LC3-I levels and an increase in LC3-II levels. Our research showed that OSCC cells Cal-27 and SCC-15 also underwent autophagy under hypoxia conditions. In physiological conditions, Beclin-1 and Bcl-2 form a complex compound that results in inhibiting the activation of the autophagy pathway. It has been confirmed that BNIP3 is the target molecule of HIF-1α. Under hypoxia conditions, the expression of HIF-1α significantly augments, which upregulates BNIP3, and BNIP3 interacts with Bcl-2 or Bcl-XL and ultimately forms heterodimer, which will prevent the binding of Bcl-2 to Beclin-1, thereby the released Beclin-1 will activate the autophagy pathway [44]. As a result, HIF-1α/BNIP3/Beclin-1 signaling pathway is an important way of inducing autophagy under hypoxic conditions.

Mild hyperthermia (41–43 °C) could directly damage proteins and organelles and thus trigger cytoprotective autophagy to tolerate the cellular stresses and prolong the survival of cancer cells [45–47]. Literature reported that heat stress-induced autophagy in several types of cancer cells, such as hepatocellular carcinoma cells (SMMC7721 and Huh7), cervical cancer cell (HeLa), and lung cancer cell (A549 cell) [30, 43]. Our experimental data also indicated that hyperthermia could induce autophagy in both hypoxia and normoxic starvation microenvironments and that autophagy was further enhanced in hypoxia conditions. This might stem from the following reasons: HT-induced protein denaturation and aggregation results in the up-regulation of HSPs, which are reported to up-regulate the autophagy mediator Beclin-1 [16]. Moreover, Hyperthermia can induce oxidative stress in cells and can further augment the generation of ROS [48]. ROS is a known inducer of autophagy and apoptosis. It has been reported that ROS acted on the complex formed by Beclin-1 and anti-apoptotic Bcl-2 homologs such as Bcl-2 and Bcl-xL. Moreover, this complex repressed the pro-autophagic activity of Beclin-1 and ROS could induce the dissociation of autophagy molecules Beclin 1 and Bcl-2, thus activating the Beclin1-induced autophagy pathway, increasing the expression of LC3-II, thereby initiating autophagy-associated pathways [49, 50]. ROS is also reported to upregulate the activity of HIF1-α which facilitates the activation of the HIF1-α/BNIP3/Beclin1 autophagy signaling pathway. In our study, the expressions of Beclin1 and LC3-II significantly increased when cells were treated with hypoxia alone and increased even further when exposed to hyperthermia, which supported the above mechanism. Namely, HIF1-α/BNIP3/Beclin1 autophagy signaling pathway was activated under hypoxia conditions and hyperthermia further enhanced the activation of the pathway by secreting ROS.

And our research results demonstrated that cell migration was inhibited and cell apoptosis rate was significantly augmented when untreated or hypoxia-treated cells were exposed to hyperthermia. Existing literature shows that HMGB1 release occurs passively as cell permeability breaks down upon necrosis [51] and late stage of apoptosis [52]. And in our experimental results, the secretion of HMGB1 was upregulated by hyperthermia. Nevertheless, during tumor development and cancer therapy, HMGB1 has been reported to play paradoxical roles in promoting both cell survival and death by regulating multiple signaling pathways. It has been demonstrated HMGB1 increases pro-survival autophagy in a Beclin1-dependent way during chemotherapy [53]. Moreover, it has been demonstrated ATG5-mediated autophagy pathway promoted the secretion of HMGB1 in starvation and lipopolysaccharide treatment, and ROS signaling was required in this process [54]. What is more, several of the secondary messengers, such as cytosolic free calcium and ROS can regulate HMGB1 secretion [53]. We also demonstrated that both hyperthermia and hypoxia facilitated the secretion of HMGB1. Furthermore, previous research has confirmed that secreted HMGB1 activated receptors for advanced glycation end products and Toll-like receptor-4 and induced autophagy in skeletal muscle [55].

Recently, more and more researchers have paid attention to the manipulation of autophagy to enhance the efficacy of cancer therapy. YC-1 is a guanylate cyclase-activator and inhibitor of HIF-1α [56] while 3-MA is a key drug in studying autophagy, which can block autophagy [57]. Therefore, we used YC-1 and 3-MA to examine the effect on autophagy. In the present study, we found the use of YC-1 significantly downregulated the expression of HIF-1α induced by CoCl_2_, although the exact mechanism is uncertain. We observed that inhibition of HIF-1α significantly suppressed BNIP3 expression and the administration of YC-1 and 3-MA alone or in combination significantly downregulated the expression of autophagy-related genes LC3II, Beclin1, and HMGB1, and increased the expression level of P62 when exposed to mild hyperthermia, hypoxia, and nutrition deficiency microenvironment. Furthermore, we found that YC-1 and 3-MA treatment suppressed cell migration and increased cell apoptosis in Cal-27 and SCC-15 cell lines, which suggested that hyperthermia and hypoxia-induced a protective effect of autophagy by activating the HIF-1α/BNIP3/Beclin1 pathway and by stimulating the secretion of HMGB1, and autophagy act as a survival mechanism to alleviate hyperthermia and hypoxia injury. But even more importantly, this result could be reversed by the use of autophagy inhibitors and by blocking HIF-1α. Given the clinical application, when therm-chemotherapy was used in combination, HT-related elevated blood perfusion also supported higher intra- and peritumoral drug concentrations, changed the tumor microenvironment to improve the efficacy of chemotherapy, while chemotherapy could effectively inhibit HT-induced autophagy to increase cell apoptosis. Autophagy and apoptosis are often inseparable and highly interactional. The research found that specifically blocking autophagy enabled ROS to increase significantly in malignant tumor cells. Thus, autophagy can aggravate the apoptosis of tumor cells [58]. The previous study shows that both apoptosis and autophagy are activated in response to metabolic stress [59], and accumulating evidence reveals that autophagy and apoptosis can cooperate, antagonize or assist each other, thus influencing the differential fate of the cell [60].

## Conclusions

Although HT is considered to be a promising cancer treatment regimen, cellular damage caused by heating could be repaired and reversed by the production of HSPs and autophagy process in hypoxia and starving environment, resulting in incomplete cell necrosis and attenuating the effects of HT therapy. In this study, we demonstrated that exposure to hypoxia and hyperthermia could induce autophagy in the OSCC cells Cal-27 and SCC-15. This process could be reversed by the use of an autophagy inhibitor and by blocking HIF-1α. In summary, our findings might benefit a further understanding of the biological effects of thermo-chemo-therapy on cancer cells, and we believed that inhibition of autophagy might be a useful and promising therapeutic strategy to enhance the therapeutic effect of HT in hypoxia and nutrient deficiency tumor environments. In addition, further research on animal models is required in the future.

## Material and methods

### Human OSCC clinical samples

The study was approved by the Ethics Committee of Yantai Yuhuangding Hospital and written informed consent was provided by all patients. OSCC and pare-cancer (tumor margin adjacent normal tissues) were obtained from 80 patients with primary OSCC, including 56 men and 24 women, aged 37–86 years, who underwent surgical resection of the tumor at the Yantai Yuhuangding Hospital between August 2015 and April 2017. None of the patients had received any chemotherapy or radiotherapy before excision. All samples were confirmed by pathological examination. The histological grade and tumor stage were assigned according to the World Health Organization (WHO) [61] and the International Union against Cancer classification system [62].

### Immunohistochemistry (IHC)

The expression levels of HIF-1α and Beclin1 were analyzed by IHC. Briefly, antigen retrieval was performed by incubating the sections in 10 mM citric acid buffer (pH 6.0) at 100 °C for 15 min. Subsequently, sections were dewaxed in xylene at room temperature and rehydrated in a descending ethanol series (absolute ethanol for 5 min, 95% ethanol for 5 min, 90% ethanol for 5 min, and 80% ethanol for 5 min). Following three washes with PBS-Tween (0.05% Tween-20 in PBS), the sections were blocked by 5% BSA (Sangon Biotech Co., Ltd., China) in TBS for 45 min at room temperature. The sections were subsequently incubated at 4 °C overnight with rabbit anti-HIF-1α or anti-Beclin1 polyclonal antibody diluted in 3% BSA/TBS solution (1:100). The slides were washed with PBST and incubated with an HRP-conjugated goat anti-rabbit IgG secondary antibody (1:1000) at room temperature for 45 min. The slides were subsequently stained with 3,3′-diaminobenzidine tetrahydrochloride at room temperature for 10 min, and then counterstained with 0.5% Harris’ hematoxylin at room temperature for 5 min. Finally, the sections were dehydrated with ethanol (80% ethanol for 5 min, 90% ethanol for 5 min, 95% ethanol for 5 min, and absolute ethanol for 5 min), dried as well as mounted with neutral balsam. The images were screened using a microscope (magnification, ×100 and ×400). For scoring staining intensity, the expression level of HIF-1α and Beclin1 in TSCC tissues were evaluated using a numerical scale (−, negative; +, positive; ++, moderate positive; +++, strong positive; Fig. 1).

### Cell culture

The human OSCC cell lines Cal-27 and SCC-15 were cultured in RPMI 1640 medium (Biological Industries, Israel), supplemented with 10% fetal bovine serum (FBS) (Gibco, Grand Island, USA) and 100 U/ml penicillin-streptomycin (Invitrogen, Carlsbad, USA), and maintained in a humidified atmosphere at 37 °C with 5% CO_2_. The following reagents were used in cell culture: 3-Methyladenine (3-MA) (Selleck, USA), 3-(5′-hydroxymethyl-2′-furyl)−1-benzylindazole (YC-1) (Sigma, USA), Cobalt chloride (CoCl_2_) (Solarbio, Beijing, China), which were dissolved with DMSO (Selleck, USA).

### Cell cytotoxicity and cell viability assay

CCK-8 assay (Sangon, Shanghai, China) was used to detect the cytotoxic effect of different drug treatments on cancer cells and the 50% inhibitory concentration (IC50) of the drug was calculated. OSCC cells were plated into 96-well plates with a density of 5 × 10^3^ cells/well, supplied with 100 µL complete growth medium. After 24 h incubation, cells were exposed to CoCl_2_, YC-1, and 3-MA at the concentrations of 25 μM to 200 μM, 10 μM to 100 μM, 0.5 μM to 150 μM, respectively. Untreated cells were used as control. At each time point, the cells were washed and incubated with 100ul RPMI 1640 plus 10 μL CCK-8 solution at 37 °C for 3 h. Subsequently, the absorbance was measured at 450 nm with a microplate reader (Bio Tek Instruments, Inc., USA). Each experiment was performed at least in triplicate. Dose-response curves were established to determine the IC50 values for CoCl_2_, YC-1, and 3-MA in the two OSCC cell lines.

### Establishment of hypoxic environment and heat treatment

A hypoxic environment was established by exposing cells to a serum-free medium with IC50 of CoCl_2_. Hyperthermia treatment was performed by partially submerging a cell culture flask in a thermostatically controlled circulating water bath (Shanghai Yiheng Scientific Instrument Co, LTD, China). Cells were treated at 42 ± 0.1 °C for 60 min and cool down to 37 °C in less than 5 min.

### RNA extraction and qRT-PCR

Total RNA from cells was extracted with trizol reagent (Sangon, Shanghai, China). cDNA was synthesized by using PrimeScript^TM^ RT Master Mix Kit (Takara, Japan). qRT-PCR was performed on a StepOne™ Real-Time PCR System (Applied Biosystems, USA) with an SYBR Premix Ex Taq Kit (TaKaRa, Japan). According to the manufacturer’s instructions, the PCRs were conducted at 95 °C for 30 s, followed by 40 cycles of 95 °C for 3 s, and 60 °C for 30 s. All reactions were performed in triplicate. The 2^−ΔΔCT^ method was applied to calculate the relative fold change of gene expression. All results were normalized to GAPDH. Primer sequences (Sangon, Shanghai, China) were listed below: HIF-1α: up:5′–AGTTCCGCAAGCCCTGAAAGC–3′, down:5′–GCAGTGGTAGTGGTGGCATTAGC–3′; Beclin1:up:5′–ATCTAAGGAGCTGCCGTTATAC–3′, down:5′–CTCCTCAGAGTTAAACTGGGTT-3′; BNIP3: up:5′–AGGGCTCCTGGGTAGAACT–3′; down:5′–CTCCATTATAAATAGAAACCGAGGC-3′; GAPDH: up:5′–GCCACATCGCTCAGACACCA–3′; down:5′–TTCCCGTTCTCAGCCTTGAC–3′; LC3II: up:5′–GTCAGCGTCTCCACACCAATCTC–3′; down:5′–TCCTGGGAGGCATAGACCATGTAC–3′; SQSTM1 (P62): up:5′–TGATTGAGTCCCTCTCCCAGATGC–3′; down:5′–CCGCTCCGATGTCATAGTTCTTGG–3′.

### Western blot

Cells were collected and lysed in RIPA buffer (Beyotime, Shanghai, China). Total protein was quantified using the BCA kit (Beyotime, Shanghai, China) according to the manufacturer’s instructions. Subsequently, the proteins were separated by 10–12% SDS-PAGE and transferred to PVDF membranes (Bio-Rad Laboratories, Inc.), which were blocked with 10% nonfat milk and incubated with primary and secondary antibodies. Afterward, the protein bands were visualized with an ECL detection kit (Millipore, Burlington, MA) and analyzed using Image-Pro Plus 6.0 software (Media Cybernetics, Inc). The following antibodies were used: Beclin-1 (Cat. no.: NB500-249, Selleck, USA, 1:1000), HIF-1α (Cat. no.: BF8002, BI, Israel, 1:1000), BNIP3 (Cat. no.: D221876, 1:1000), LC3B (Cat.no.: D163557,1:1500), SQSTM1/p62 (Cat. no.: sc-48402, 1:1500), mouse anti-β-actin monoclonal antibody (Cat. no.: MA1-744, 1:3000), mouse anti-GAPDH monoclonal antibody (Cat. no.: SC-47724, Santa, USA, 1:2000), HRP-conjugated goat anti-mouse IgG antibody (Cat. no.: 31430, 1:3000) and HRP-conjugated goat anti-rabbit IgG antibody (Cat. no.: 314600, 1:3000). Antibodies were all obtained from Invitrogen, USA unless otherwise stated.

### Cell migration assay

Cells were seeded in 6-well plates and cultivated until 100% confluence. In the serum-free RPIM 1640 medium, 100 μM CoCl_2_ was added to simulate a hypoxia environment, and cells were treated separately with 50 μM 3-MA and/or 25 μM YC-1 combined with heat treatment for 1 h in a 42 °C water bath. Then the cells were scraped with 200 μL of the pipette tip and washed with PBS three times. At 0 and 24 h after incubation in serum-free medium, the images of wound healing were captured using an inverted microscope (magnification, ×400). The area of each wound was quantified using Image-Pro Plus 6.0 software (Media Cybernetics, Inc.). The cell migration rate (%) was calculated as follows: [(Area of the wound at 0 h − Area of the wound at 24 h)/Area of the wound at 0 h] × 100%.

### Flow cytometry analysis of apoptosis

Cell apoptosis was detected by flow cytometry using the FITC-AnnexinV/PI Apoptosis Assay Kit (BD, USA) following the manufacturer’s instructions. Briefly, the cells (5 × 10^5^ cells/well) that treated with chemotherapy (YC-1 and 3-MA) and hyperthermia (42 °C heat treatment) were harvested and centrifuged at 500×*g* for 5 min at room temperature, then washed twice with PBS and resuspended in 500 μL binding buffer solution at a density of 1 × 10^5^ cells/mL. The cells were subsequently stained with 5 μL FITC Annexia V and 5 μL PI using at room temperature for 15 min in the darkness. Apoptotic cells were analyzed using a CytoFLEX flow cytometer and CytExpert software (version 2.0; Beckman Coulter, Inc.) within 1 h.

### Enzyme-linked immunosorbent assay (ELISA)

HMGB1 is passively released by necrotic tissues or actively secreted by stressed cells. To measure extracellular HMGB1, supernatants of cell culture were collected and centrifuged at 8000×*g* for 5 min and immediately analyzed by enzyme-linked immunosorbent assay (ELISA) (Solarbio, Beijing, China) according to the manufacturer’s instructions. The levels of HMGB1 in chemotherapy (YC-1 and 3-MA) and hyperthermia (42 °C heat treatment)-treated Cal-27 and SCC-15 cells that were in hypoxia and normoxia condition were determined using ELISA kits (Ilerite Biotechnology Co, China), in line with the manufacturer’s protocol.

### Statistical analysis

Statistical analysis was performed using the GraphPad Prism version 8.0 (GraphPad Software, La Jolla, CA). Data were presented as the mean ± standard deviation, comparisons between groups were performed by using Student’s *t*-test or a one-way ANOVA, followed by a Tukey’s post hoc test for multiple comparisons. A χ^2^ test was used to determine the association between the expression levels of HIF-1α, Beclin1 respectively, and OSCC clinical and histopathological features. *p* < 0.05 was considered a statistically significant difference Table 2.

**Table 2:** Expression difference of HIF-1α and Beclin1 in OSCC tissues and para cancer tissues (*n*, %).

Group	Number	HIF-α protein expression		χ^2^	*P*	Beclin1 protein expression		χ^2^	*P*
		negative	positive			negative	positive		
Cancer tissues	80	23 (28.75%)	57 (71.25%)	25.42	P < 0.01	29 (36.25%)	51 (63.75%)	13.23	*P* < 0.05
Para cancer tissues	80	49 (61.25%)	21 (26.25%)			52 (65.00%)	28 (35.00%)		

## Supplementary information

The table of IC50 of Cocl2, YC-1, and 3-MA.

supplementary figure s-fig1

supplementary figure s-fig2

supplementary figure s-fig3
