# Supplementary material for: Combined effects of hyperthermia and chemotherapy on the regulate autophagy of oral squamous cell carcinoma cells under a hypoxic microenvironment
Source: Cell Death Discov. 2021 Aug 31;7:227. doi: 10.1038/s41420-021-00538-5 (PMC8408236; doi:10.1038/s41420-021-00538-5)
Supplement: Supplementary file 1 — The table of IC50 of Cocl2, YC-1, and 3-MA. [file 41420_2021_538_MOESM1_ESM.docx]

**[supplementary](javascript:;)**[**materials**](javascript:;)

Table1 Expression difference of HIF-1α and Beclin1 in OSCC tissues and para cancer tissues (n, %)

| Group | Number | HIF-α protein expression | | χ^2^ | *P* | Beclin1 protein expression | | χ^2^ | *P* |
| --- | --- | --- | --- | --- | --- | --- | --- | --- | --- |
|  |  | negative | positive |  |  | negative | positive |  |  |
| Cancer tissues | 80 | 23 (28.75%) | 57 (71.25%) | 25.42 | P<0.01 | 29 (36.25%) | 51 (63.75%) | 13.23 | P<0.05 |
| Paracancer tissues | 80 | 49 (61.25%) | 21 (26.25%) |  |  | 52 (65.00%) | 28 (35.00%) |  |  |

Table 3 Cell viability after 24 h of Cocl_2_ was detected by CCK-8 assay

| group | 5uM | 25uM | 50uM | 100uM | 150uM | 200uM |
| --- | --- | --- | --- | --- | --- | --- |
| Cal-27 | 79.767±1.673 | 74.170±1.307 | 67.193±1.376 | 56.533±1.785 | 40.83±0.753 | 21.753+0.420 |
| Scc-15 | 81.777±1.724 | 73.05±1.197 | 67.687±0.556 | 55.280±1.997 | 34.053±0.523 | 16.153±2.077 |

Table 4 Cell viability after 24 h of YC-1 was detected by CCK-8 assay

| group | 5uM | 10uM | 20uM | 50uM | 80uM | 100uM |
| --- | --- | --- | --- | --- | --- | --- |
| Cal-27 | 66.747±1.272 | 53.797±1.146 | 41.930±2.763 | 29.257±0.882 | 21.627±0.332 | 10.810±0.357 |
| Scc-15 | 79.333±0.757 | 69.320±1.995 | 58.123±1.595 | 48.487±1.147 | 28.710±2.933 | 16.237±2.011 |

Table 5 Cell viability after 24 h of 3-MA was detected by CCK-8 assay

| group | 2.5uM | 5uM | 25uM | 50uM | 80uM | 100uM |
| --- | --- | --- | --- | --- | --- | --- |
| Cal-27 | 95.107±2.633 | 84.607±0.618 | 76.137±1.146 | 57.297±1.679 | 36.533±1.676 | 24.997±1.061 |
| Scc-15 | 87.203±2.038 | 79.683±1.337 | 68.630±0.726 | 48.720±2.623 | 40.37±1.776 | 29.367±1.606 |

**The cause of Cobalt chloride was chosen to simulate hypoxia**

Low oxygen‐induced hypoxia is the optimal hypoxia model. However, the problem faced by our laboratory is we don’t have a hypoxia chamber or at least a CO_2_ incubator with a regulated level of oxygen suitable for carrying out the experiments. Because a larger hypoxia incubator that allows the media to be changed and cells to be manipulated in a continuous hypoxic environment is expensive. We only have CO_2_ incubator in our lab, and the use of a CO_2_ incubator has the problem that not all types of experiments can be performed because oxygen re‐enters the chamber at each opening. The induction of “hypoxic conditions” using CoCl_2_ allows the experimenter to open the culture plate/dish/flask many times while maintaining the stabilization of HIF.

Therefore, one of the most commonly used hypoxia mimics is CoCl_2_‐induced chemical hypoxia. CoCl_2_ strongly stabilizes HIF‐1α and HIF‐2α under normoxic conditions. Compared to low oxygen‐induced hypoxia and the use of other hypoxia mimics, the stabilization of HIF‐1α and HIF‐2α is sustained for several hours. Hence, this model allows users a wider time window to manipulate and analyze their samples under normoxic conditions. It is currently accepted that Co^2+^ substitutes Fe^2+^ in prolyl hydroxylases (PHDs), the key enzymes that link O2 concentration to the degradation of HIF under normoxic conditions[1].

The relationship between the concentration of Cocl_2_ and induced hypoxia

In general, HIF‐1α/2α stabilization is observed since 2 hours, with a maximum at 12‐48 hours and between 100 and 300 μM. It has been observed that above 200-300μM, and depending on the cell type used, CoCl_2_ exerts a toxic effect that leads to cell death oxidative DNA damage by ROS combined with inhibition of DNA repair[2, 3]. However, apoptosis, autophagy or necrosis cell death has been described for different cell lines as result of CoCl_2_ use[3, 4].

For this reason, Co^2+^ has been widely used in studies of the genetic response modulated by HIF‐1 and HIF‐2 factors that mimic the hypoxic transcriptional response. Several works have proposed different mechanisms by which Co^2+^ stabilizes and promotes HIF‐1α/2α subunit protein accumulation. These proposals are addressed below.

In contrast to dimethyloxaloglycine (DMOG) and deferoxamine (DFO), Co^2+^ has shown a better effect on HIF‐1α and ‐2α stabilization[5].

The mechanism by which cobalt chloride simulates hypoxia

1. Replacement hypothesis

The replacement hypothesis proposes that the mechanism responsible for HIF‐1α stabilization is the inhibition of PHD activity through displacement of Fe^2+^ by Co^2+^, Ni^2+^ or Mn^2+^ in the PHD active site.

1. Ascorbate and/or Fe^2+^ oxidation hypothesis

In the PHD reaction, ferrous iron (Fe^2+^) is oxidized to Fe^3+^. Ascorbic acid or ascorbate is an essential cofactor that maintains Fe^2+^ in its reduced state. Oxidized ascorbate is unable to reduce Fe^3+^ to Fe^2+^ in the catalytic sites of PHD and FIH, thus inhibiting the activity of each enzyme

3. Direct interference of Co^2+^ between von Hippel‐Lindau protein binding and hypoxia inducible factor‐α protein.Subsequent in vitro experiments showed that cobalt: (1) inhibits the hydroxylation of Pro564 within the ODD of HIF‐2α; (2) stabilizes cellular HIF‐2α by occupying the pVHL‐binding domain; and (3) inhibits the interaction between pVHL and HIF‐2α even when HIF‐2α is hydroxylated[6].

4. Regulation of hypoxia inducible factor‐1α mRNA by cobalt

It has been observed that CoCl_2_ upregulates HIF‐1α mRNA. An increase in HIF‐1α transcription upon exposure to Co^2+^, with an initial peak at 2‐4h, was observed in human Hep3B [7]. In U87MG and T98G cells, the use of flavopiridol, a transcriptional elongation factor (P‐tEFb) inhibitor, corroborated that cobalt increases the transcription of HIF‐1α mRNA [8]. Dail et al [9] demonstrated that there is a correlation between Co^2+^ and HIF‐1α mRNA in a dose‐ and time‐dependent manner.

5. CoCl_2_ modulates the translational upregulation of hypoxia inducible factor‐1α

CoCl_2_ activates ERK1/2 and PI3K signaling pathways, leading to the translational upregulation of HIF‐1α. The observed CoCl2 effect is blocked by inhibitors of ERK1/2 and PI3K [10, 11]

6. CoCl_2_‐induced hypoxia inducible factor‐1α stabilization by factor inhibiting hypoxia inducible factor and acetyltransferase downregulation.

CoCl_2_ inhibits histone acetylation and increases the methylation of histone H3 at lysine 9. This effect may play a role in the downregulation of these two important genes with a negative effect on HIF‐1α activation [12].

The similarities between low oxygen‐induced hypoxia and CoCl_2_‐induced chemical hypoxia.

The use of CoCl_2_ increases HIF‐1α/2α in a dose‐dependent manner, similar to that observed in hypoxia. Several representative genes that encode proteins such as vascular endothelial growth factor (VEGF) and erythropoietin (EPO), as well as some genes that encode glycolytic enzymes, such as aldolase, phosphoglycerate kinase 1 and pyruvate kinase M, and glucose transporters, such as GLUT1, are induced by CoCl_2_‐increased HIFs[13, 14]. It has also been demonstrated that CoCl_2_ reproduces the same responses as hypoxia on cell death.

Reference:

1. Muñoz-Sánchez, J. and Chánez-Cárdenas, M.E. (2019) The use of cobalt chloride as a chemical hypoxia model. J Appl Toxicol 39 (4), 556-570.

2. Lison, D. et al. (2001) Update on the genotoxicity and carcinogenicity of cobalt compounds. Occup Environ Med 58 (10), 619-25.

3. Simonsen, L.O. et al. (2012) Cobalt metabolism and toxicology--a brief update. Sci Total Environ 432, 210-5.

4. Kanaji, A. et al. (2014) Cytotoxic effects of cobalt and nickel ions on osteocytes in vitro. J Orthop Surg Res 9, 91.

5. Borcar, A. et al. (2013) Metabolic preconditioning of mammalian cells: mimetic agents for hypoxia lack fidelity in promoting phosphorylation of pyruvate dehydrogenase. Cell Tissue Res 351 (1), 99-106.

6. Yuan, Y. et al. (2003) Cobalt inhibits the interaction between hypoxia-inducible factor-alpha and von Hippel-Lindau protein by direct binding to hypoxia-inducible factor-alpha. J Biol Chem 278 (18), 15911-6.

7. Wang, G.L. et al. (1995) Hypoxia-inducible factor 1 is a basic-helix-loop-helix-PAS heterodimer regulated by cellular O2 tension. Proc Natl Acad Sci U S A 92 (12), 5510-4.

8. Newcomb, E.W. et al. (2005) Flavopiridol downregulates hypoxia-mediated hypoxia-inducible factor-1alpha expression in human glioma cells by a proteasome-independent pathway: implications for in vivo therapy. Neuro Oncol 7 (3), 225-35.

9. Dai, Z.J. et al. (2012) Up-regulation of hypoxia inducible factor-1α by cobalt chloride correlates with proliferation and apoptosis in PC-2 cells. J Exp Clin Cancer Res 31 (1), 28.

10. Chachami, G. et al. (2004) Cobalt induces hypoxia-inducible factor-1alpha expression in airway smooth muscle cells by a reactive oxygen species- and PI3K-dependent mechanism. Am J Respir Cell Mol Biol 31 (5), 544-51.

11. Triantafyllou, A. et al. (2006) Cobalt induces hypoxia-inducible factor-1alpha (HIF-1alpha) in HeLa cells by an iron-independent, but ROS-, PI-3K- and MAPK-dependent mechanism. Free Radic Res 40 (8), 847-56.

12. Ke, Q. et al. (2005) Down-regulation of the expression of the FIH-1 and ARD-1 genes at the transcriptional level by nickel and cobalt in the human lung adenocarcinoma A549 cell line. Int J Environ Res Public Health 2 (1), 10-3.

13. Ouiddir, A. et al. (1999) Hypoxia upregulates activity and expression of the glucose transporter GLUT1 in alveolar epithelial cells. Am J Respir Cell Mol Biol 21 (6), 710-8.

14. Semenza, G.L. et al. (1994) Transcriptional regulation of genes encoding glycolytic enzymes by hypoxia-inducible factor 1. J Biol Chem 269 (38), 23757-63.
