# Supplementary figures and images for: Combined effects of hyperthermia and chemotherapy on the regulate autophagy of oral squamous cell carcinoma cells under a hypoxic microenvironment
Source: Cell Death Discov. 2021 Aug 31;7:227. doi: 10.1038/s41420-021-00538-5 (PMC8408236; doi:10.1038/s41420-021-00538-5)

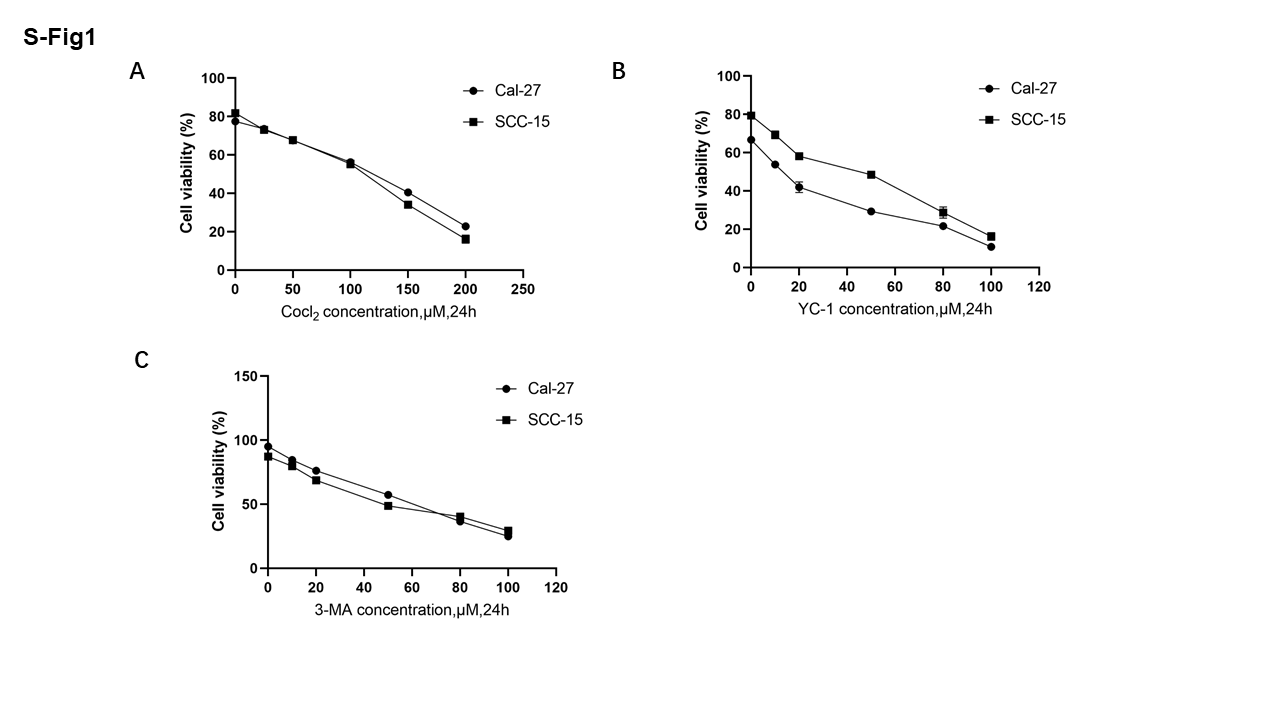

Supplement: Supplementary file 2 — supplementary figure s-fig1 [file 41420_2021_538_MOESM2_ESM.tif]

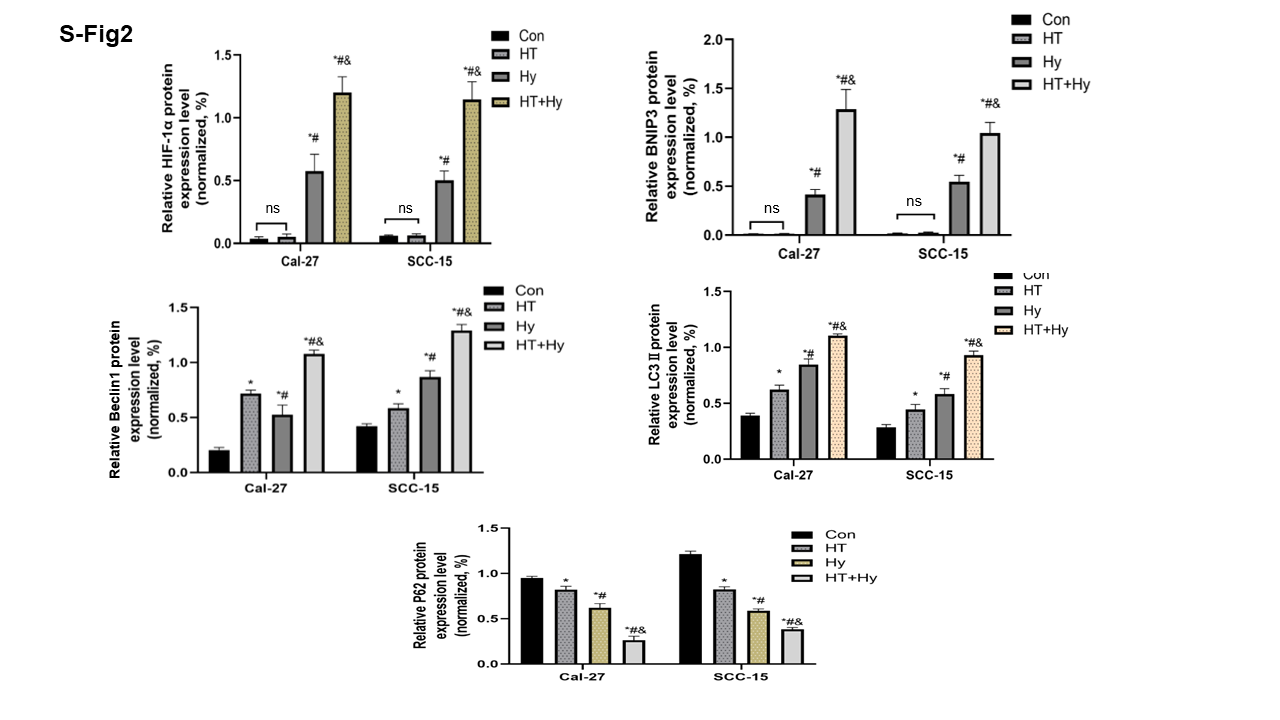

Supplement: Supplementary file 3 — supplementary figure s-fig2 [file 41420_2021_538_MOESM3_ESM.tif]

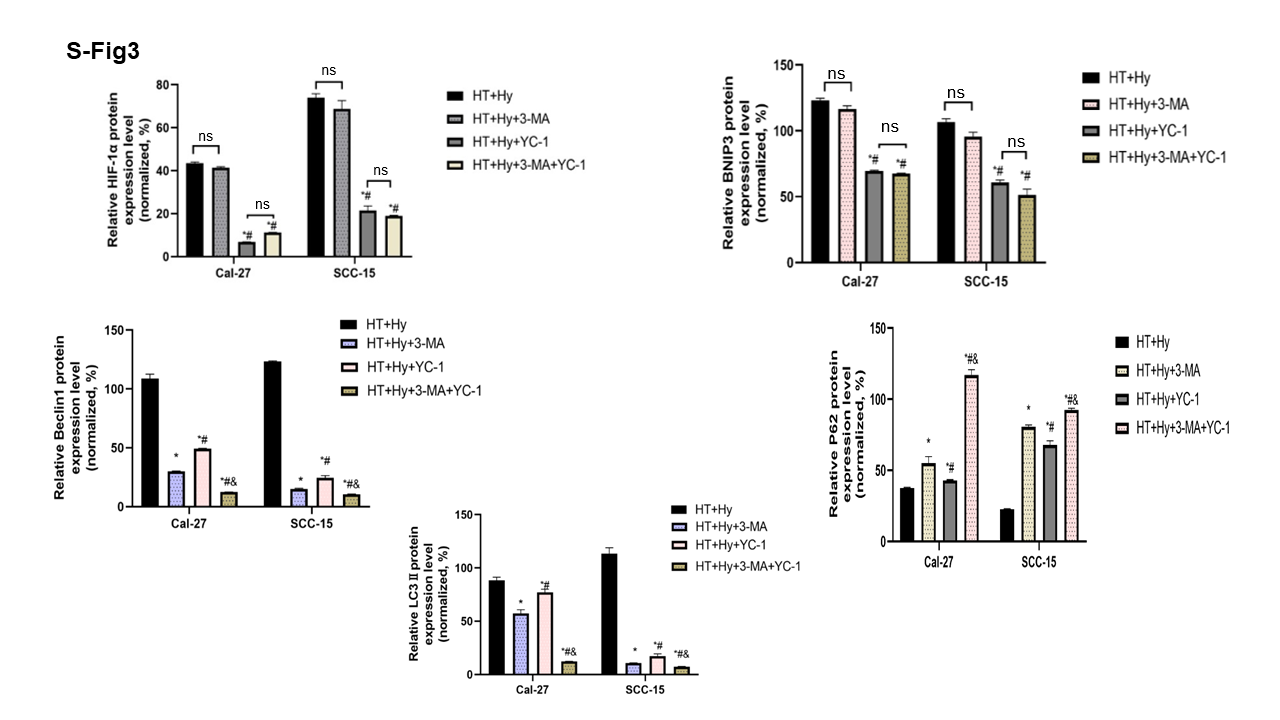

Supplement: Supplementary file 4 — supplementary figure s-fig3 [file 41420_2021_538_MOESM4_ESM.tif]
